# Transcatheter closure of paravalvular leaks: state of the art

**DOI:** 10.1007/s12471-016-0918-3

**Published:** 2016-11-22

**Authors:** I. Cruz-Gonzalez, J. C. Rama-Merchan, J. Rodríguez-Collado, J. Martín-Moreiras, A. Diego-Nieto, M. Barreiro-Pérez, P. L Sánchez

**Affiliations:** 1 Cardiology Department, University Hospital of Salamanca, IBSAL, Salamanca, Spain; 2 Cardiology Department, Hospital of Merida, Badajoz, Spain

**Keywords:** Paravalvular leak, Prosthetic heart valve, Heart failure, Haemolytic anaemia, Amplatzer Vascular Plug

## Abstract

Paravalvular leak (PVL) is a serious complication after surgical valve replacement or after transcatheter aortic valve replacement. Approximately 1–5% of PVLs can lead to serious clinical consequences, including congestive heart failure and/or haemolytic anaemia. For years, surgical re-intervention has been considered the treatment of choice for symptomatic patients with PVLs. However, surgical re-intervention is associated with a high risk of morbidity and mortality. Transcatheter PVL (TPVL) closure is a less invasive alternative to surgical re-intervention. The safety and feasibility of TPVL closure has been confirmed in several registries and a meta-analysis.

In this review, we discuss the clinical implications and diagnosis of PVLs, technical considerations for TPVL, execution of the procedure and assessment of the results.

## Introduction

Paravalvular leak (PVL) is a serious complication after surgical valve replacement or after transcatheter aortic valve replacement (TAVR) [1, 2]. The incidence of PVLs after surgical valve replacement varies in different studies, ranging from 2–10% in the aortic position and from 7–17% in the mitral position [1, 3, 4]. Significant (moderate or severe) paravalvular regurgitation has been reported in up to one-quarter of patients following TAVR [5].

Although most PVLs are small, remain asymptomatic and follow a benign clinical course, larger PVLs with serious clinical consequences, such as heart failure, severe haemolytic anaemia or endocarditis, occur in 1–5% of patients who have undergone surgical valve replacement, with most occurring in association with prosthetic mitral valves [3, 4, 6, 7].

For years, surgical re-intervention has been considered the treatment of choice for symptomatic patients with PVLs. It was shown that the overall mortality was lower in the surgical re-intervention group compared with the group treated conservatively [4]. However, the operative mortality of surgical treatment of PVL is still high. Long-term outcomes remain suboptimal in these challenging patients, especially in the presence of multiple previous surgical re-interventions and associated co-pathologies [8]. Transcatheter PVL (TPVL) closure has emerged as a safe, effective and less invasive alternative to surgical re-intervention [9–12] with durable symptom relief in selected patients [10].

In this review, we discuss the clinical implications and diagnosis of PVLs, technical considerations for TPVL, execution of the procedure and assessment of results.

## Diagnosis of paravalvular leak

**Fig. 1:**
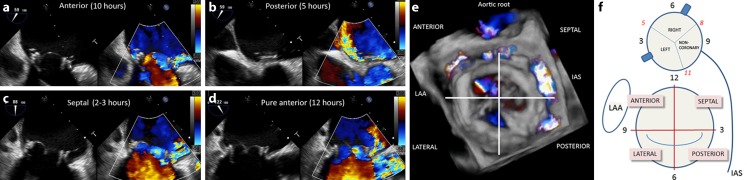
Mechanical prosthetic valve with four PVLs (**a–d** show different positions of leaks in 2D and colour echocardiography). To locate and assess different leaks on 2D echo needs a careful search. Instead, colour 3D-TEE makes this an easier task (**e**), taking into consideration the lower temporal and spatial resolution. It is necessary to refer PVL in relation to anatomic reference points (**f**). Taking the surgeon’s view from left atrium, leaks would be referred to in hours, as a clock, taking mitro-aortic curtain as 12 h and/or by position segments. *LAA* left atrial appendage, *IAS* interatrial septum

**Fig. 2:**
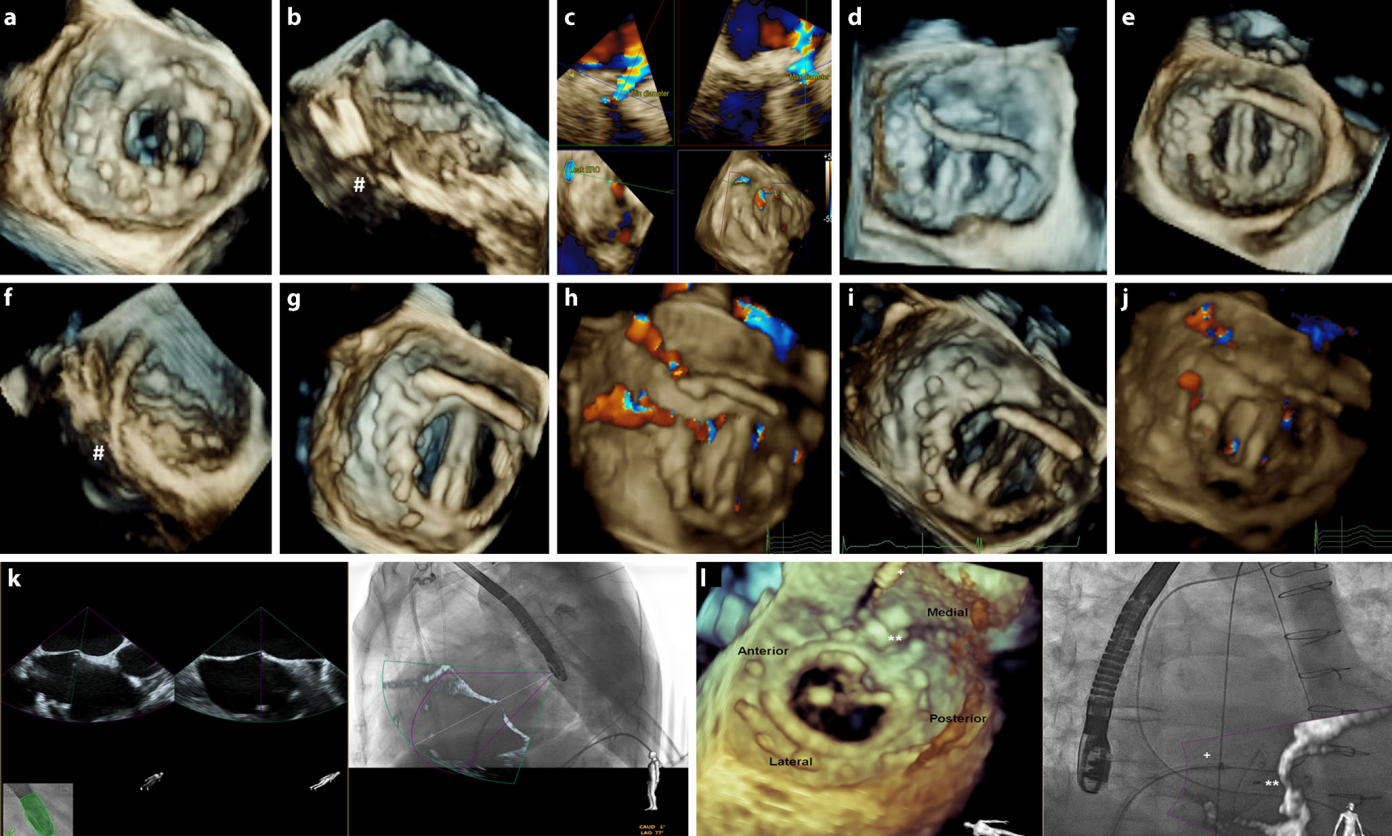
3D-TEE during TPVL closure over mechanical prosthetic mitral valve with the leak partially closed with three devices and a residual defect at 10–11 h (**a**). Cropping 3D-volume (**b**) is possible to visualise the leak tunnel (#). PVL assessment using multiplanar reformatting over 3D-colour (**c**). After transseptal puncture, guide and sheath are moved to left atrium (**d**) and crossed through PVL (**e** and **f**, same cropping as **b**). Device deployment should be monitored to detect any disturbance (**g**) and reduction in regurgitation (**h**) prior to device release (**i** and **j**). Fusion imaging could be useful, for example, during transseptal puncture (**k**) or crossing the PVL with the guide wire (**l**)

In some cases, PVL diagnosis can be challenging. Often the first signs of suspicion are the presence of an abnormal murmur in the physical examination and the evidence of significant haemolytic anaemia in blood tests. Blood cultures to eliminate endocarditis should be considered. In cases of suspected PVL, patient evaluation must be followed by an echocardiography study to confirm the diagnosis. Two-dimensional (2D) transoesophageal echocardiography (TEE) is very sensitive in accurately identifying the presence of PVL [13]; however, in many cases, assessing the number, shape and location of the defects with 2D-TEE is practically impossible [14]. Three-dimensional (3D)-TEE allows better definition of the PVLs, making this technique the gold standard for PVL evaluation (Fig. 1). 3D-TEE is especially useful for mitral PVLs, being almost mandatory for a correct diagnosis [15–17]. It is recommended that mitral PVL location be reported in a clockwise format from a surgeon’s perspective or ‘surgical view’ (Fig. 1; [15, 16, 18]). Also, the aortic PVL location should be reported in a clockwise format [9]. TEE is crucial during the TPVL closure procedure, while 3D-TEE plays an essential role in the selection of the closure device, for guiding the procedure, and assessment of the results (Fig. 2; [15, 16]). Furthermore, fusion imaging techniques provide a valuable extra help during the procedure, i. e. EchoNavigator automatically fuses live 3D-TEE and live X‑ray in real time, so it can intuitively guide the procedure in the 3D space.

Due to the complexity of some defects, other image modalities such as computed tomography (CT) and cardiac magnetic resonance (CMR) could be very useful [16, 19]. Recently introduced tools such as the 3D-Heart Navigator, which combine 3D-CT images with an overlay of the live X‑ray fluoroscopy information, can also assist during the procedure.

## Transcatheter techniques for TPVL closure

### Crossing the PVL

**Fig. 3:**
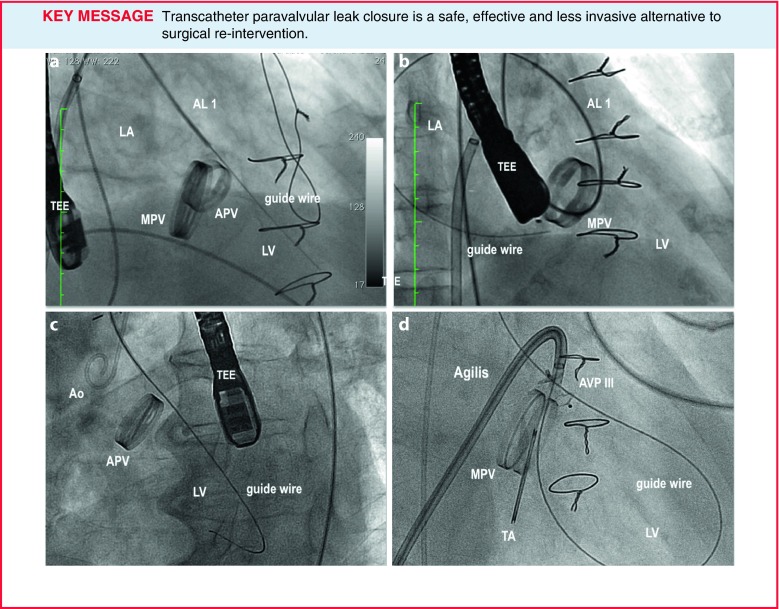
Technical approaches to TPVL closure. **a** Mitral PVL closure using an antegrade transseptal approach. **b** Mitral PVL closure using a retrograde approach. **c** Aortic PVL closure using a retrograde approach. **d** Mitral PVL closure using a deflectable sheath. *APV* aortic prosthetic valve, *MPV* mitral prosthetic valve, *Ao* Aorta, *AL* Amplatzer left, *LA* left atrium, *LV* left ventricle, *AVP* Amplatzer Vascular Plug, *TEE* transoesophageal echocardiography, *TA* tricuspid annuloplasty

*Mitral TPVL closure: *In the antegrade approach (Fig. 3), once a successful transseptal puncture is made, a diagnostic catheter (e. g., Judkins right) is advanced into the left atrium. After that, a hydrophilic guidewire (e. g., Terumo guidewire, Terumo Medical Corporation) is often used to cross the mitral PVL and in most cases an arteriovenous loop is established in the aorta, alternatively an extra-support wire can be placed in the left ventricle. Finally, a delivery sheath is advanced from the venous access over the loop or extra-support wire and the device is deployed. Before releasing, the disc movement in mechanical valves should be confirmed. It can be very helpful to use a safety guidewire to avoid having to re-cross the PVL. Sometimes (e. g. in septal PVL or if the angle between the transseptal puncture and the defect is unfavourable) it can also be very helpful to use a deflectable catheter (e. g., Agilis, St Jude Medical), (Fig. 3; [11]).

In the retrograde approach, a hydrophilic guidewire (e. g., Terumo guidewire, Terumo Medical Corporation) over a catheter (e. g., Judkins right) is often used to cross the PVL from the left ventricle to the left atrium. After crossing, an arteriovenous wire loop is often created in the left atrium; therefore, a transseptal puncture is needed. Finally, the delivery sheath is advanced from the venous access and the device is deployed (Fig. 3).

Transapical access can be a good alternative for mitral PVL closure (especially for posterior or septal defects or patients with mitro-aortic mechanical valves) [9]. The main advantages of this access are the often less difficult wiring of the PVL and less resistance to cross the PVL.

*Aortic PVL closure: *The retrograde approach is used for closure of most aortic PVLs (Fig. 3). The defect is usually crossed using a hydrophilic guidewire over a catheter (e. g. Amplatzer Left-1). After crossing, this guidewire is exchanged for a stiffer wire (e. g., Amplatz Super-stiff^TM^, Boston Scientific). Finally, the delivery sheath is advanced and the device is deployed. In some cases an arterio-arterial loop can be established for added support. The technique for PVL closure after TAVR is similar to the technique for aortic PVLs after surgical valve replacement [20].

### Choosing the sheath

There are different sheaths that can be used for delivering the device. The size of the leak dictates the device, which dictates the sheath/guide used for delivery (Table 1). In most of the cases the Amplatzer TorqVue (St Jude Medical, St. Paul, MN, USA) sheaths are used. If there is difficulty in crossing the leak, other options such as the Terumo Destination Guiding Sheath (Terumo, Japan) or the Sheathless Eucath (Ashahi Intec, Japan) should be considered as they provide a larger lumen with smaller outer diameter. It should be taken into account that the largest Occlutech Paravalvular Leak Devices (PLD, Occlutech, Helsingborg, Sweden) need a 10 F sheath. In certain cases, where TPVL closure cannot be completely achieved with a single device, multiple devices can be deployed using simultaneous sheaths or sequentially using different techniques [21].

**Table 1:** Amplatzer and Occlutech devices: main characteristics

**Amplatzer devices**				
	*Size* *(central waist)*	*Length* *(central waist)*	*Difference between disc and central waist*	*Sheath size (Fr)*
ASO	4–40 mm (every 1 mm up to 20 mm, >20 mm, every 2 mm)	3–4 mm	8–12 mm (ASO 4–10) 10/14 mm (ASO >11) 10/16 mm (ASO >34)	6–12
AmVSDO	4–18 mm (every 2 mm)	7 mm	8 mm	5–9
ADO	5–16 mm distal end and 4–14 mm proximal	5–8 mm	4 mm (ADO 5/4–8/6) 6 mm (ADO 10/8–16/14)	5–7
AVP II	3–22 mm (every 2 mm)	6 mm	–	4–7
AVP III	Long axis: 4–14 mm Short axis: 2–5 mm	2–5 mm	2 mm	4–7
AVP IV	4–8 mm	10–13.5 mm	–	4–5
**Occlutech devices**				
	*Length of the distal disc*	*Length of the proximal disc*	*Length × Width*	*Size (Fr)*
Occlutech PLD Rectangular W^a^				
4 W 6 W 8 W 10 W 12 W 14 W 16 W 18 W	11.5 14 16.5 19 21 24 26.5 28.5	10 12.5 15 17 19 22 24.5 26.5	4 × 2 6 × 3 8 × 4 10 × 4 12 × 5 14 × 6 16 × 8 18 × 10	6 6 7 8 9 9 10 10
Occlutech PLD Rectangular T^b^				
5 T 7 T 10 T 12 T	13 16 19 21	11.5 14 17 19	–	6 7 8 9
Occlutech PLD Square W^a^				
4 W 5 W 6 W 7 W	13 14 16 17	11.5 12.5 14 16	4 × 4 5 × 5 6 × 6 7 × 7	6 6 6 7
Occlutech PLD Square T^b^				
3 T 5 T 7 T	11.5 14 17	10 12.5 16	–	6 6 7

*ASO* Amplatzer Septal Occluder, *AmVSDo* Amplatzer Muscular VSD Occluder, *ADO* Amplatzer Duct Occluder, *AVP* Amplatzer Vascular Plug, *PVL* paravalvular leak

^a^W (Waist)

^b^T (Twist)

### Choosing the device

**Fig. 4:**
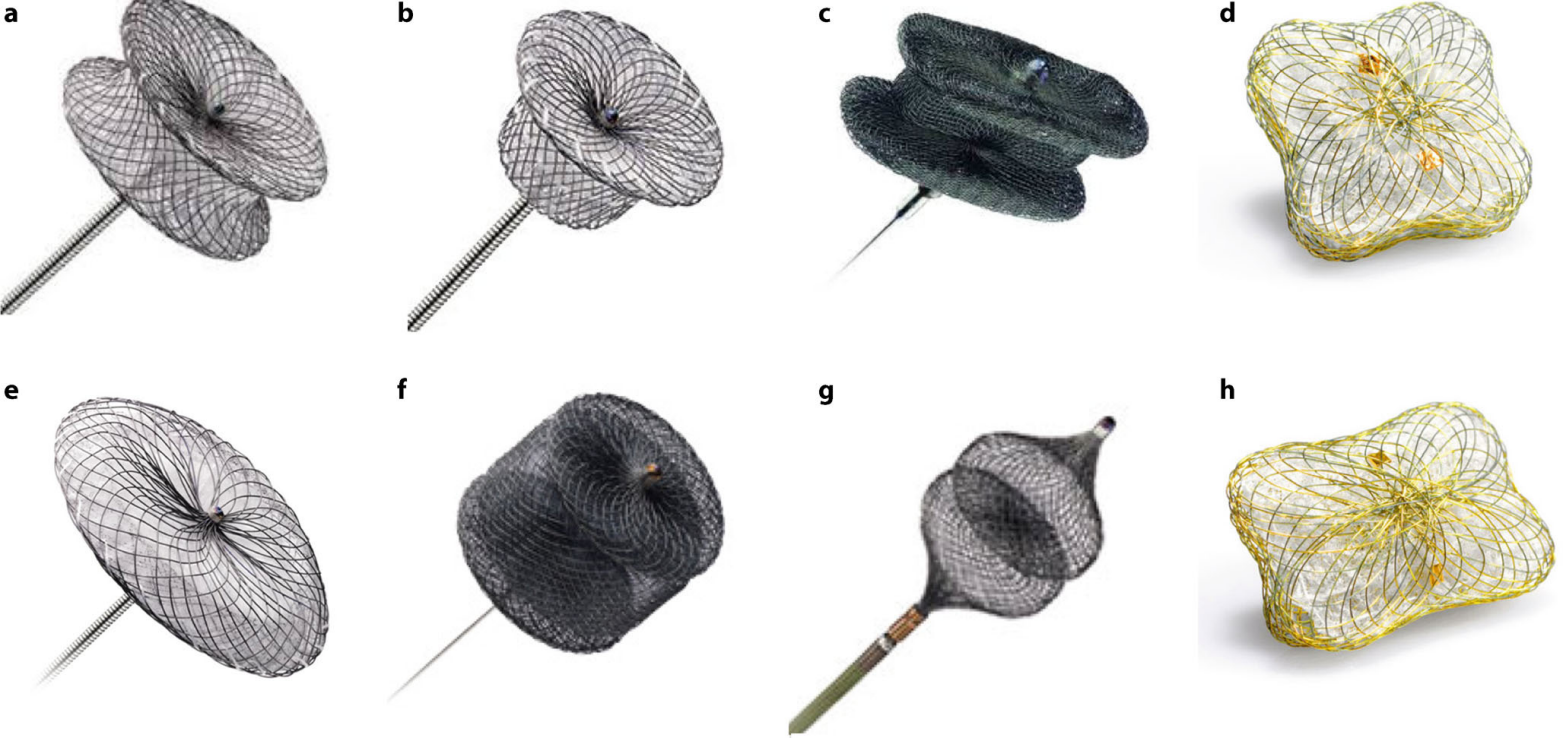
PVL closure devices. **a** Amplatzer Muscular VSD Occluder. **b** Amplatzer Duct Occluder. **c** Amplatzer Vascular Plug III. **d** Occlutech PLD (square-shaped design). **e** Amplatzer Septal Occluder. **f** Amplatzer Vascular Plug II. **g** Amplatzer Vascular Plug IV. **h** Occlutech PLD (rectangular-shaped design)

Most of the devices used today have not been designed, tested, or approved for PVL closure and they are used ‘off-label’ for this purpose (Fig. 4). PVLs are variable in size and shape with many being crescentic and serpiginous, rather than cylindrical, which makes it extremely difficult for one device to fit in all PVLs. Amplatzer devices (St. Jude Medical, St. Paul, MN, USA) are currently the most used for TPVL closure [9, 11, 22–24]. The Amplatzer Vascular Plug (AVP) II is widely used in the USA while in Europe the most frequently used device is the AVP III. At this time, the only device specifically approved to TPVL closure by the European Commission is the Occlutech PLD [25].

For a small cylindrical PVL, we will often use an AVP II or PDA occluder. For an oval PVL, an AVP III occluder is preferred. For a small PVL with significant angulation and a small neck (typically after TAVR), an AVP IV occluder can be considered. Sizing of these devices usually comes from 2D- and 3D-TEE measurements. Angiography can also be useful for aortic PVL. The PLD rectangular-shaped device is also recommended to cover crescent-shaped defects while avoiding interference with the valve.

## Outcomes and complications of PVL closure procedures

The safety and feasibility of TPVL closure has been confirmed in several registries and a meta-analysis [9, 10, 12, 23, 26]. Reported technical and clinical success ranged from 77–86% and 67–77% respectively (Table 2; [9, 10, 12, 23, 26, 27]). Also, successful TPVL closure has been associated with a lower cardiac mortality rate compared with failed closure (260 patients; OR 0.08; 95% CI 0.01–0.90), a positive tendency toward lower all-cause mortality (311 patients; OR 0.52; 95% CI, 0.09–1.74) and a superior functional class improvement or improved haemolytic anaemia compared with failed TPVL closure (267 patients; OR 9.95; 95% CI, 2.10–66.73). Complication rates are low at experienced centres (Table 3; [9, 10, 22, 23, 28, 29]). It has recently been shown in a single-centre non-randomised study that percutaneous PVL closure results compare favourably with surgical treatment [30]. Furthermore, in 308 PVL closure procedures attempted in 259 patients in 20 centres Calvert et al. [29] reported a technical success rate of 91%, PVL improved post-procedure (*p* < 0.001) and was none (33.3%), mild (41.4%), moderate (18.6%) or severe (6.7%) at last follow-up. The mean New York Heart Association class improved from 2.7 ± 0.8 pre-procedure to 1.6 ± 0.8 (*p* < 0.001) after a median follow-up of 110 (7–452) days.

**Table 2:** Summary of main PVL closure studies

	Hein [28]	Cortés [26]	Ruiz [9]	Sorajja [11]	Noble [27]	Cruz [23]	Sánchez [22]	Calvert [29]
Patients, *n*	21	27	43	126	56	33	20	259
Mean age, years	65	63	69	67	65	71	68	67
Male, %	62	81	67	53	52	45	60	28
*Indication*								
CHF, % Haemolysis, % Both, %	38 10 52	33 11 56	16 14 60	71 7 22	61 9 30	21 3 76	55 5 40	80 16 2
*Prosthesis*								
Mechanical, *n* Bioprosthesis, *n*	– –	27 0	15 28	49 77	50 6	32 1	15 5	57 38
*Patients with*								
Mitral PVL Aortic PVL	13 8	27 0	33 10	99 27	44 12	26 7	14 6	44 48
*Approach*								
Anterograde Retrograde Both	– – –	17 – –	– – –	100 32 13	44 12 0	7 26 0	– – –	104 173 17
*Device implanted*								
AVP III, *n* AVP II, *n* ADO, *n* mVSD, *n* ASO, *n* OPLD, *n*	0 0 8 13 5 –	0 0 17 0 0 –	0 5 39 11 2 –	0 77 20 10 12 –	7 0 18 28 0 –	34 0 0 0 0 –	18 2 0 0 1 –	184 9 12 41 20 11
Technical success, %	95	62	86	91	75	94	85	91
Procedural success, %	90	37	81	76	71	91	80	NYHA improved from 2.7 ± 0.8 pre-procedure to 1.6 ± 0.8
Mean follow-up, months	13.5	3	42	11 (median)	30 (median)	3	12 (median)	3.7 (median)

*PVL* paravalvular leak, *CHF* chronic heart failure, *AVP* Amplatzer vascular plug, *ADO* Amplatzer duct occluder, *mVSD* Amplatzer muscular ventricular septal defect occluder, *ASO* Amplatzer septal occluder, *OPLD* Occlutech® Paravalvular Leak Device, *NYHA* New York Heart Association

**Table 3:** Main complications associated with PVL closure

Complication	Percentage
Device embolisation	4% [9]
Cardiac perforation	4%^a ^[9], 0% [23], 0% [11]
Death	2% [9], 1,7% [11]
Vascular complications	2% [9], 0,9% [11]
Embolic stroke	1.7% [11]
Emergency cardiac surgery for prosthetic impingement	0.9% [11]
Intracranial haemorrhage	0.9% [11]
Sepsis	0.9% [11]

^a^Transapical access

On the other hand, experience with TPVL closure after TAVR is still limited, but it could be a reasonable strategy in selected patients [2]. TPVL closure after TAVR is associated with high success rates and with an improved functional status [2]. The most used devices are the AVP II, III and IV [2].

## Medical therapy and follow-up

For patients under anticoagulation therapy this should be continued after the procedure. Dual antiplatelet therapy for at least 3 months is recommended in non-anticoagulated patients (i. e. biological prostheses). Post-procedural imaging with TEE to assess device position and residual regurgitation is recommended. The timing of a follow-up TEE varies between institutions but we recommend an initial early TEE 3 months after procedure.

## Conclusions and future directions

TPVL closure in symptomatic patients with severe PVL is a less invasive option than surgical re-intervention, with lower procedural morbidity and mortality. However, surgical re-intervention is still a valuable option for large defects or cases that cannot be treated percutaneously. To continue to improve procedural success and outcomes, new advancements in device designs are necessary. The ideal PVL closure device should meet the following criteria: (a) should conform to the often ‘irregular’ defects, (b) have low-profile deliverability, (c) be repositionable and retrievable, (d) avoid interference with prosthetic valve leaflets, (e) accomplish complete closure of the defect, (f) have a low risk of embolisation or dislodgement and (g) should not be thrombogenic. In the same way recently introduced imaging tools such as the 3D-Heart Navigator or the Echo Navigator will improve the results of this complex intervention.
